# Expiry of veterinary medicines in supply outlets in Central Uganda: prevalence, management and associated factors

**DOI:** 10.1186/s40545-023-00569-6

**Published:** 2023-05-10

**Authors:** Stevens Kisaka, Frank K. Tumwebaze

**Affiliations:** 1 College of Veterinary Medicine, Animal Resources and Biosecurity, Makerere University, Kampala, Uganda; 2 School of Public Health, Makerere University, Kampala, Uganda; 3 Ministry of Agriculture, Animal Industry and Fisheries (Government of Uganda), Kampala, Uganda; 4 Eastern and Southern African Management Institute (ESAMI), Kampala, Uganda

**Keywords:** Expiry, Veterinary medicines, Supply outlets, Uganda

## Abstract

**Background:**

Animal diseases are a danger to livestock, businesses, and public health. This is why the public and private sectors in Africa have invested immensely in the manufacture and distribution of veterinary drugs. However, veterinary drug supply chain actors still suffer losses as a result of expiration. Besides, the way expired products are managed might pose risks to human, animal, and environmental health. This study investigated the prevalence, management of, and factors associated with the expiry of veterinary medicines in supply outlets in Central Uganda.

**Methods:**

A cross-sectional study was conducted among owners and caretakers of veterinary drug supply outlets. Data were collected using self-administered, semi-structured questionnaires. The level of expiry was computed as “acceptable” (for levels reported as ≤ 5%) and “unacceptable” (for levels reported as ˃5%). Logistic regression was used to assess associations between the level of drug expiry and predictor variables.

**Results:**

A total of 168 owners/caretakers of veterinary drug supply outlets were included in this study. The majority (148/168, 88.1%) of respondents reported having experienced expiries in their outlets. Unacceptable levels of expiry were reported in 38/168 (22.6%) of the outlets. Retail outlets accounted for the majority (34/38, 89.5%) of the unacceptable levels. Powdered drugs accounted for most expiries (106/148, 71.6%). Most expiries were for drugs supplied to outlets on credit (58/124, 46.8%) and those used to treat rare diseases (26/124, 21%). Major reasons for expiry included irrational prescription, inaccurate forecasts, overstocking, dry seasons, and stocking without considering stock-at-hand. Methods of disposal of expired drugs included throwing at pits (74/168, 44.1%); returning drugs to suppliers (51/168, 30.4%); and incineration (33/168, 19.6%). Factors associated with acceptable levels of expiry included the caretaker being female [adjusted OR = 2.9, 95% CI = 1.22–5.08]; having a procurement policy [adjusted OR = 3.9, 95% CI = 1.29–4.81] and practicing first expiry, first out [adjusted OR = 6.07, 95% CI = 4.71–8.70].

**Conclusions:**

Veterinary drug expiries are common in Uganda, and environmentally unfriendly methods of disposal are widely used. Acquisition and use of inventory tracking technologies that support First-Expiry-First-Out principles as well as proper disposal of expired medicines are recommended.

## Background

Livestock production significantly contributes to human existence [1]. Approximately 1.3 billion people (i.e., 1 in 5 people worldwide) depend on livestock for their livelihood. Livestock are not only kept for food production, but also play important economic, cultural, and social roles and provide multiple functions and services [2]. The livestock sub-sector contributes about 40% of the global agricultural gross domestic product (GDP) [3]. This is why this sub-sector is central to the process of attaining Sustainable Development Goal (SDG) 2 (ending hunger, achieving food security, improving nutrition, and promoting sustainable agriculture) and those SDGs that are directly related to it like SDG 3 (good health and wellbeing). Due to the global population explosion and rapid urbanization, the demand for livestock products is also growing quickly. It is therefore not a surprise that the global biomass of livestock is estimated to be twice that of human populations [1, 4, 5]. The sub-sector is attracting multitudes of farmers, processors, and marketers who are striving to meet the rising demand.

Given the importance of livestock in human society, competent and reliable animal health services are an essential requirement for the development of the livestock sub-sector, especially in sub-Saharan Africa [6]. In Africa, animal diseases represent a major constraint to livestock development and business; for example, they impinge on not only animal productivity, but also public health [7, 8]. However, in some African countries, the state is no longer involved in the provision of clinical veterinary services or the supply of veterinary drugs [9]. In Uganda, due to the highly privatized nature of most veterinary products, handling and distribution are done by the private sector, forming a multimillion-dollar industry. Many importing and distributing firms have invested large amounts of resources in buying and distributing veterinary drugs within the country [6]. Nevertheless, there is still wastage as a result of drug expiries, which have occasioned losses to both the dealers and the end-users [10, 11].

Expired veterinary drugs not only result in economic losses to the dealers, but also have the potential to endanger the health of handlers (veterinarians and farmers), animals, and consumers of animal products. In addition, they have been indicted for environmental contamination, which has become a growing concern worldwide. Their role in spreading antimicrobial resistance, interfering with reproduction, and increasing the incidence of cancer in humans has also been well elaborated [12, 13]. This explains why laws bar outlets from dispensing such drugs in Uganda. It is illegal for any person to use an expired product for animal treatment under any conditions and those who breach this incur penalties or even possible jailing. In addition, expired stock of veterinary drugs is supposed to be dealt with following National Drug Authority guidelines [14] at the cost of the dealer, resulting in added economic losses. However, although there are over 350 registered veterinary outlets in the study area [15], little is known about the volumes of expired drugs that form part of the veterinary waste generated or about its management. If timely and safe disposal of expired drugs is absent, they may be indiscriminately dumped, thus posing the risk of environmental pollution, or even repackaged for the counterfeit market [16].

In general, stockpiles of expired pharmaceuticals usually accumulate due to poor dealer forecasts of future demand. This in turn is attributed to deficiencies in the management of a supply chain or to poor coordination between suppliers and dealers as a result of weaknesses in the drug distribution mechanism and management system. It has been noted that the inherent structural and technical dysfunction in the management of a pharmaceutical supply chain can promote the accumulation of large quantities of expired pharmaceuticals in Uganda [16]. On the human medicines side, the contributing factors to expiries in the supply chain have been identified as abandonment of stock monitoring, lack of knowledge and skills on elementary tools that prevent expiry, clinicians not participating in quantifying the medicines in hospitals, quantification based on profits and incentives, overstocking, as well as vertical programs that undertake vertical procurement [17].

The challenge of expired medicine staying within the supply chain or posing public health risks is of global concern. In Bhutan, for fear of future shortages, it is not uncommon to find veterinarians and para-veterinarians keeping expired drugs for sale and use in times of deficiency [18]. In Malawi, the practice of selling products that were about to expire or had expired at a low price to avoid losses is a common occurrence [19]. Likewise, in Ethiopia, some private veterinary drug shop owners and public veterinary pharmacy attendants do not dispose of expired drugs for the same reasons. Worse still, burning expired veterinary drugs is the most popular method of disposal in Ethiopia [20]. Similarly, in Pakistan, some veterinarians disposed of expired drugs in the communal garbage [21] just like it is in the USA [22]. In Brazil, the disposal of leftover and expired veterinary drugs in the urban solid waste collection network and their direct release into the water used in aquaculture have been fairly well described [23].

Nonetheless, there was limited information on how expired veterinary medicines in supply outlets were being handled in Uganda. In addition, the specific contributing factors to such expiries were not well documented. Therefore, this study aimed at investigating the extent, management of, and factors associated with the expiry of veterinary medicines in supply outlets in Uganda. The information obtained may contribute to better supply chain management in the sector, thus promoting the profitability of businesses while enhancing the efficient use of veterinary medicines.

## Methods

### Study site

The study was conducted in selected districts of central Uganda. The study area has approximately 350 registered veterinary outlets, and these are either importers, distributors, or retailers. The study area was chosen because it has the highest concentration of veterinary drug outlets, which may be explained by its proximity to Kampala, the capital city of Uganda, where major drug wholesalers carry out their business [11, 15]. In addition, the area is the most industrialized in Uganda and includes the Great Kampala Metropolitan Area, which is home to 70% of the country’s manufacturing plants and contributes over a third of the overall national gross domestic product (GDP) [24]. In some places in the study area, approximately 90% of drug retailers do not have specialized training in veterinary medicine, while 72% of veterinary practitioners are not conversant with the veterinary drug policies of the country [11].

### Study design and population

This was a cross-sectional study involving owners and caretakers of the veterinary outlets. Where the business was owned by more than one person or had more than one caretaker, the one to be recruited was chosen using a simple random approach. All the outlets were involved in retail, although 34 of them doubled as wholesalers and 21 were importers. It is mandatory for all outlets to have an operating license obtained following the licensing guidelines of the National Drug Authority [25]. However, outlets without displayed valid operating licenses at the time of the study were excluded.

### Sample size and sampling procedures

The study used a census approach, in which all 201 veterinary drug outlets in the selected districts were involved. The drug outlets are accessible and operate as formal businesses; hence, they could be identified for data collection. The study site was chosen purposively because of its high concentration of veterinary outlets. A consecutive sampling approach was used to recruit the study respondents. This was done by following a list of all veterinary outlets registered in selected districts at the National Drug Authority (NDA). In this register, the location, type (importer, wholesaler, distributor or retailer), owner, and telephone contacts were extracted. For each district, the place where the outlet is situated was traced with the help of the contact details in the register by calling the corresponding telephone number for directions if the outlet could not be located using the primary information from the register. Upon arriving at the outlet, the main person in charge of the business was identified and consented to participate in the study. The outlet was then marked off the list, and the researchers proceeded to the next outlet to collect the data.

### Study variables

The dependent variable was the level of expiry of veterinary drugs. Drug expiration is the date after which a drug might not be suitable for use as manufactured. The shelf life of a drug can be determined by checking its pharmaceutical packaging for an expiration date. The respondents were asked to calculate the value of the expired stock realized in the past year as a percentage of the total value of the stock held in that year. When the respondent recorded that they experienced expiries of 5% or less in the past year, it was taken that such an outlet had “acceptable levels”. Levels of expiry that were beyond 5% were taken to be “unacceptable”. This was based on the human drug expiry standards in Uganda after noting that the veterinary standards do not exist in the country [26].

Explanatory variables that were studied included:

- Sociodemographic and business characteristics: location of business (by district); gender (male or female); age (in years); position in business (pharmacist, stores manager, manager, owner, salesperson or other); experience in the veterinary drug business (in years); cost of stock that is being held on average (below 200 million, between 200 and 500 million and above 500 million Uganda shillings); source of stock (Uganda, Europe, Asia, USA, and Africa); and highest level of education attained.
- Organizational factors: type of outlet (retailer or wholesaler); dedicated inventory personnel (yes/no); experience with drug expiries (yes/no); written procurement policy (yes/no); and type of drugs usually expire (mention).
- Inventory management factors: techniques such as FIFO (yes/no) and FEFO (yes/no); receiving near-expiry (yes/no); storage conditions for example storage space (shelf, store, or shelf and store); warehousing training (yes/no); logistics management information system (yes /no), stock record availability (yes/no); stock record utilization (yes/no); stock taking frequency (number of times per year); and presence of an automated records system (yes/no).
- Reasons for expiries: overstocking (yes/no); need for bigger profit margins thus big sizes of purchases (yes/no); drugs dumped/ pushed by suppliers (yes/no); not making accurate forecasts (yes/no); procurement being done irrespective of present stock (yes/no); dry seasons when veterinary drugs are not on demand (yes/no); quarantines that slow down demand for veterinary drugs in critical areas of the country (yes/no); slowed demand in neighboring countries, e.g., due to instability there (yes/no); irrational prescribing of drugs by veterinarians hence underuse of certain medicines (yes/no); lack of data on stock hence inaccurate forecasts (yes/no); supplier’s incentives (e.g., discounts) that lead to stocking drugs that are not on demand (yes/no); not having a timetable to inspect stock for expiry dates (yes/no); poor records and filing (yes/no); not regularly monitoring inventory levels (yes/no); no specific personnel to do inventory management (yes/no); time between placing order and delivery of drugs is too long (yes/no); and long customs processes causing delays hence drugs are got when they are near expiry (yes/no).

### Data collection and quality control

The information about the extent of expiries and related factors and practices was collected using a self-administered, semi-structured questionnaire that was developed based on literature review. Six research assistants were recruited, and a pair was assigned to a particular cluster of districts. The research assistants were recruited based on their demonstrated experience in data collection and possession of a bachelor’s degree. The research assistants were trained and oriented to the study purpose, rationale, and data collection instruments, as well as ethical considerations. After the training, the research team pretested the questionnaires in five veterinary drug outlets in Mbarara district, in western Uganda. Results from the pretests were used to refine the research instrument, share experiences from the field, and draw lessons for the actual data collection exercise.

Actual data collection was undertaken through physical visits to the business premises/veterinary drug outlets. After recruitment of the respondent, the questionnaire was given to the participant to answer the indicated questions. The research assistant stayed with the respondent and offered assistance whenever the former asked for it. Where the in-charge was reported to be away, the questionnaire was left behind and a phone call was made to the individual to secure an appointment for a time that they would be available. At that particular time, a research assistant was sent to that particular outlet to have the respondent fill out the questionnaire.

### Data management and analysis

Completed questionnaires were entered in EpiData software (EpiData version 2.0, Epidata, 2005, Denmark) and analyzed in SPSS software (SPSS Statistics version 22.0, IBM, 2017, USA). Descriptive analysis was done, and the distribution of variables was summarized as proportions/percentages for categorical variables. Comparisons between groups were made using the Chi-square test (or Fisher’s exact test, where appropriate). Logistic regression was used to determine the association between the outcome (drug expiry) and the predictor/explanatory variables. The odds ratios for factors associated with drug expiry were recorded, and all variables independently associated with drug expiry at bivariate analysis (*p* < 0.25) were considered for the multi-variable model to evaluate the effect of those variables on expiry. Having identified variables at simple logistic regression with a *p*-value ≤ 0.25, both backward and forward stepwise selection methods were used to build the multiple logistic regression model while assessing the model variables for significance at a *p*-value ≤ 0.05 and 95% level of confidence. The model explained the variables/factors associated with the level of expiry of veterinary drugs.

### Ethical considerations

The study was conducted with approval by the Eastern and Southern African Management Institute (ESAMI) Research and Ethics Committee (ESAMI/DA/40E/2021). It was explained to the respondents that the study posed no direct risks and that they would benefit if the information obtained in the study is used to improve the governance of the veterinary pharmaceuticals in the country. Written informed consent was obtained from the respondents before commencement of the study. The respondents’ data were anonymized for purposes of maintaining confidentiality. The data were stored and managed in ways that upheld research norms.

## Results

### Characteristics of study participants and veterinary drug outlets

Overall, 168 of 201 respondents participated in the study, representing a response rate of 83.6%. The main reason for nonparticipation was the inability of the caretakers to obtain permission from the outlet owners to participate in the study. Of the 168 who participated, the majority were female (100/168, 59.5%). Nearly half of the respondents were sales personnel (92/168, 54.8%). The majority of respondents (98/168, 58.3%) had a certificate as the highest level of education attained, while 25/168 (14.9%) possessed a minimum of a degree. Most participants resided and had their businesses located in Wakiso district (60/168, 35.7%); one-third in Kampala city (44/168, 26.2%); and only six resided in Buikwe district (6/168, 3.6%). Overall, the majority of the participants interviewed were retailers (137/168, 81.6%). Almost half of the outlets in the study required that only the owner place orders for new stock and not any other person (82/168, 48.8%). The median number of years spent in the veterinary drug sector for the in-charges was 4 (interquartile range, IQR: 5) years. The sociodemographic characteristics, stratified by the type of supplier, are indicated in Table 1.

**Table 1:** Sociodemographic characteristics of the 168 of study participants in the veterinary drug outlets

Variable	Categories	Frequency *n* (%)	Type of supplier	
			retail *n* (%) 137 (81.6)	Wholesale *n* (%) 31 (18.4)
District	Mukono	24 (14.3)	24 (100)	0
	Wakiso	60 (35.7)	60 (100)	0
	Kampala	44 (26.2)	15 (34.1)	29 (66)
	Mpigi	20 (11.9)	18 (90)	2(10)
	Buikwe	6 (3.6)	6 (100)	0
	Luwero	14 (8.3)	14 (100)	0
Sex	Male	68 (40.48)	56 (82.4)	12 (17.7)
	Female	100 (59.5)	81 (81)	19 (19)
Position	Pharmacist	14 (8.3)	8 (57.1)	6 (42.9)
	Stores manager	1 (0.6)	1(100)	0
	Manager	35 (20.8)	29 (82.9)	6 (17.1)
	Owner	26 (15.5)	22 (84.6)	4 (15.4)
	Salesperson	92 (54.8)	7 7 (83.7)	15 (16.3)
Education level	Certificate	98 (58.3)	98 (100)	0
	Diploma	45 (26.8)	38 (84.4)	7 (15.6)
	Degree and above	25 (14.9)	1 (4)	24 (96)
Years spent in the business	≤ 5 years	108 (64.3)	91 (84.3)	17 (15.7)
	6–10 years	39 (23.2)	31 (79.5)	8 (20.5)
	˃10 years	21 (12.5)	15 (71.4)	6 (28.6)

This table shows the sociodemographic characteristics, stratified by the type of supplier. Most participants resided and had their business located in Wakiso district (60/168, 35.7%). Overall, majority of the participants interviewed were retailers (137/168, 81.5%)

In terms of the supply outlets that were included in this study, most had stock that was valued at < 200 million Uganda shillings (USD 55,000) at the time of this research. However, most of the outlets with < 200 million Uganda shillings were retailers, while those with bigger amounts of stock were wholesalers. Slightly less than half of the outlets (82/168, 48.8%) had a written procurement policy, while just a quarter (41/168, 24.4%) had an electronic inventory management system. Conspicuously, electronic inventory systems were more common among wholesalers than retailers (*p*˂0.001), as shown in Table 2.

**Table 2:** Characteristics of the 168 veterinary drug outlets included in the study

Variable	Categories	Frequency *n* (%)	Type of supplier		*p*-value (ChiSq/exact Pr)
			Retail *n* (%)	Wholesale *n* (%)	
Cost of stock, in UGX (USD)***	< 200 M (55,000)	114 (67.9)	109 (95.6)	5 (4.4)	< 0.001*
	200 M–500 M (55,000–137,000)	32 (19.1)	19 (59.4)	13 (40.6)	
	> 500 M (137,000)	22 (13.1)	9 (40.9)	13 (59.1)	
Source of stock	Uganda	158 (94.1)	133 (84.2)	25 (15.8)	< 0.001*
	Asia, Africa and Europe	10 (5.9)	4 (40)	6 (60)	
Procurement policy	Yes	82 (48.8)	70 (85.4)	12 (14.6)	0.213
	No	86 (51.2)	67 (77.9)	19 (22.1)	
Practice FIFO	Yes	91 (54.5)	84 (92.3)	7 (7.7)	< 0.001*
	No	76 (45.5)	52 (68.4)	24 (31.6)	
Practice FEFO	Yes	145 (86.3)	177 (80.7)	28 (19.3)	1.000**
	No	23 (13.7)	20 (86.9)	3 (13.1)	
Electronic system	Yes	41 (24.4)	24 (58.6)	17 (41.5)	< 0.001*
	No	127 (75.6)	133 (89.0)	14 (11.0)	

This table shows the characteristics of veterinary drug outlets that were included in the study. Most of the outlets had stock that was valued at < 200 M Uganda shillings (USD 55,000) at the time of this research. Slightly less than half of the outlets (82/168, 48.8%) have a written procurement policy while just a quarter (41/168, 24.4%) had an electronic inventory management system

^*^Statistical significance at *p* ≤ 0.05 using **Chi-square tests; **Fisher’s exact test probability value; ***UGX = Uganda shillings; USD = United States Dollars

### Prevalence of and reasons for expiration of veterinary medicines in supply outlets

The majority (148/168, 88.1%) of respondents had experienced expiries of veterinary medicines in their outlets in the past year. The majority of outlets (130/168, 77.4%) had an acceptable level of expiry (i.e., 5% and below), while 38/168 (22.6%) were classified as having an unacceptable level of expiry.

In terms of expiry status, there were more outlets without a procurement policy in place among those that had unacceptable levels of expiry compared to those with acceptable expiries (˂0.001). Similarly, those outlets that did not practice either First-In-First-Out (FIFO) or First-Expiry-First-Out (FEFO) had higher levels of unacceptable expiry compared to those that had acceptable levels (*p* ˂0.001). Further, those with less experience in the sector were more likely to have unacceptable levels than those with more experience (*p* = 0.038). The details of other factors/characteristics by level of expiry are shown in Table 3.

**Table 3:** Characteristics of veterinary drug outlets and in-charges by expiry status in central Uganda

Variable	Categories	Frequency *n* (%)	Expiry levels		*p*-value (ChiSq/Exact Pr)
			Acceptable	Unacceptable	
Type of outlet	Retailer	137 (81.6)	103 (75.2)	34 (24.8)	0.233*
	Wholesale	31 (18.5)	27 (87.1)	4 (12.9)	
Cost of stock, in UGX (USD)***	< 200 M (55,000)	114 (67.9)	86 (75.4)	28 (24.6)	0.565
	200 M–500 M (55,000–137,000)	32 (19.1)	27 (84.4)	5 (15.6)	
	> 500 M (137,000)	22 (13.1)	17 (77.3)	5 (22.7)	
Source of stock	Uganda	158 (94.1)	125 (79.1)	33 (20.9)	0.944
	Asia, Africa and Europe	10 (5.9)	5 (50.5)	5 (50.5)	
Procurement policy	Yes	82 (48.8)	76 (92.7)	6 (7.3)	˂0.001**
	No	86 (51.2)	54 (62.8)	32 (37.2)	
Practice FIFO	Yes	91 (54.5)	80 (87.9)	11 (12.1)	˂0.001**
	No	76 (45.5)	49 (64.5)	27 (35.5)	
Practice FEFO	Yes	145 (86.3)	123 (84.8)	22 (15.2)	˂0.001**
	No	23 (13.7)	7 (30.4)	16 (69.6)	
Electronic system	Yes	41 (24.4)	33 (80.5)	8 (19.5)	0.584
	No	127 (75.6)	97 (76.4)	30 (23.6)	
Sex	Male	68 (40.48)	44 (64.7)	24 (35.3)	0.001**
	Female	100 (59.5)	86 (86.0)	14 (14.0)	
Education level	Certificate	98 (58.3)	73 (74.5)	25 (25.5)	0.558
	Diploma	45 (26.8)	37 (82.2)	8 (17.8)	
	Degree and above	25 (14.9)	20 (80.0)	5 (20.0)	
Years spent in the business	≤ 5 years	108 (64.3)	90 (83.3)	18 (16.7)	0.038**
	6–10 years	39 (23.2)	25 (64.1)	14 (35.9)	
	˃10 years	21 (12.5)	15 (71.4)	6 (28.6)	

This table shows the characteristics of veterinary drug outlets and in-charges by expiry status. There were more outlets without a procurement policy in place among those that had unacceptable levels of expiry compared to those with acceptable expiries (˂0.001). Similarly, those outlets that did not practice either First-In-First-Out (FIFO) or First-Expiry-First-Out (FEFO) had more levels of unacceptable expiry compared to those that had acceptable levels (*p* ˂0.001)

^*^Fisher’s exact test value; **Statistical significance at *p* ≤ 0.05; ***UGX = Uganda shillings; USD = United States Dollars

**Fig. 1:**
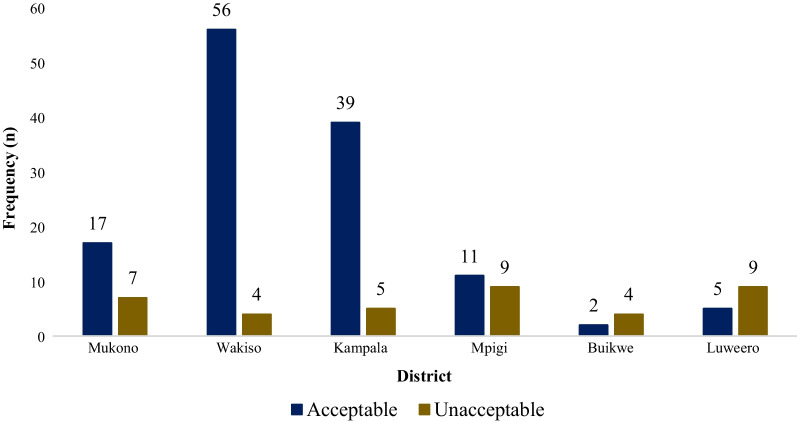
Extent of expiry of veterinary medicines in supply outlets by district in central Uganda

In terms of location, there were statistically significant differences (*p* < 0.001) in the expiry levels of the outlets, with Luweero and Mpigi having the highest levels of expiries, both at 9/38 (23.7%). The fewest expiries were experienced in Wakiso and Buikwe, both at 4/38 (10.5%), as shown in Fig. 1.

When the type of drugs that often expired was assessed, it was found that powders expired in the majority of outlets (106/148, 71.6%), followed by injectables (17/148, 11.5%), while the least mentioned were vaccines (10/148, 6.8%). The majority of those that experienced the expiry of injectables specified that antibiotics expired most frequently (24/29, 82.8%). Still, 49% of respondents that had experienced expiries describe the expiry of veterinary medicines in their outlets as being common. Similarly, 31% thought expiries were very common, while 20% described them as rare. Table 4 describes the expiries that are experienced in the drug outlets.

**Table 4:** Characteristics of expiry for the veterinary drugs in the 168 outlets in central Uganda

Characteristic	Category	Frequency	Percent
Experience expiries	No	20	11.9
	Yes	148	88.1
	Total	168	100
Type of drugs that expire	Injectable	17	11.5
	Powders	106	71.6
	Vaccines	10	6.8
	Combination of above	15	10.1
	Total	148	100
Specify injectable that expire	Antibiotics	24	82.8
	Vitamins	3	10.3
	Others	2	6.9
	Total	29	100
Frequency of expiry	Very common	46	30.9
	Common	73	49.0
	Rare	30	20.1
	Total	149	100

This table shows the characteristics of expiry for the veterinary drugs in the 168 outlets that were included in the study. Powders expired in the majority of outlets (106/148, 71.6%) followed by injectables (17/148, 11.5%) while the least mentioned were vaccines (10/148, 6.8%)

**Fig. 2:**
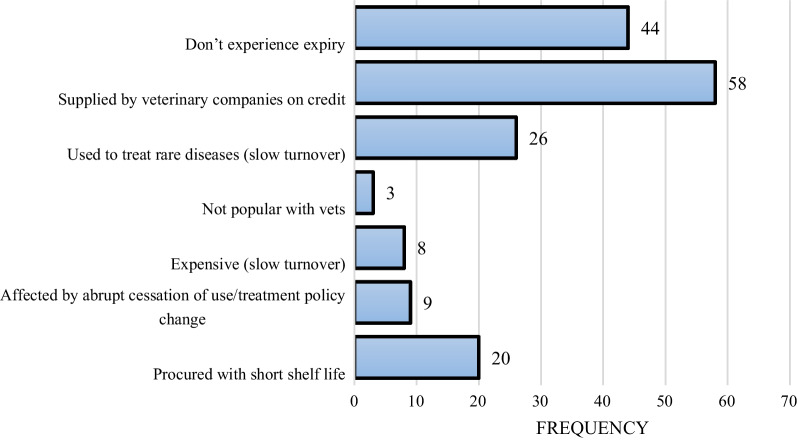
Characteristics of drugs that often expire in selected outlets in central Uganda

Study participants who experienced expiries in their outlets described the characteristics of the medicines with frequent expiry. The majority (58/124, 46.8%) of the expired drugs were those supplied by veterinary companies on credit and also used to treat rare diseases, which have slow turnover (26/124, 21.0%). Figure 2 shows the detailed list of characteristics of the drugs that expire.

When asked why there were expiries in their outlets, the majority of respondents attributed it to: irrational prescription (133/168, 79%); lack of accurate forecasting (114/168, 68%); overstocking of commodities (104/168, 62%); dry seasons; lack of adequate data on stock; procurement of commodities irrespective of the available stock (92/168, 55%); among others. Table 5 provides the details of the reasons for the expiry, together with corresponding frequencies and percentages.

**Table 5:** Reasons for expiry of veterinary medicines in selected outlets in central Uganda

Characteristic	Category	Frequency	Percent
Overstocking	No	64	38.1
	Yes	104	61.9
Need for bigger profits	No	91	54.17
	Yes	77	45.83
Lack of personnel to do inventory management	No	109	64.88
	Yes	59	35.12
Dumped drugs by supplier	No	90	53.57
	Yes	78	46.43
No accurate forecasts	No	54	32.14
	Yes	114	67.86
Procurement irrespective of present stock	No	76	45.24
	Yes	92	54.76
Dry seasons	No	65	38.92
	Yes	102	61.08
Quarantines	No	98	58.33
	Yes	70	41.67
Slow demand in neighboring countries	No	138	82.14
	Yes	30	17.86
Irrational prescription	No	35	20.83
	Yes	133	79.17
Lack of data on stock	No	70	41.67
	Yes	98	58.33
Suppliers incentives to purchasing big quantities	No	79	47.31
	Yes	88	52.69
Poor records and filing	No	83	50.0
	Yes	83	50.0
No regular monitoring	No	70	41.67
	Yes	98	58.33
Long customs processes	No	156	92.86
	Yes	12	7.14
Suppliers incentives	No	79	47.31
	Yes	88	52.69
Time between ordering and delivery	No	157	93.45
	Yes	11	6.55
No timetable to inspect expiry dates	No	84	50.0
	Yes	84	50.0

This table shows the reasons for expiry of veterinary medicines as given by the respondents. Majority of the respondents attributed expiries to; irrational prescription (133/168, 79%); lack of accurate forecasting (114/168, 68%); overstocking of commodities (104/168, 62%); dry seasons; lack of adequate data on stock; procurement of commodities irrespective of the available stock (92/168, 55%); among others

### Management practices for expired veterinary medicines in central Uganda

When asked what they do with expired drugs, the majority (74/168, 44%) mentioned that they throw them away, i.e., dump them at the pits. Of these, 53/74 (71.6%) were retailers, and the rest were wholesalers. Additionally, 51/168 (30%) said that they returned the expired drugs to the suppliers and did not know what happened to them later. There were exclusively retailers who were being supplied by the wholesalers. Notably, 10/168 (6.0%) burn the expired drugs at open garbage pits, while 33/168 (19.6%) use incineration services as advised by the National Drug Authority.

Further, respondents mentioned several strategies that they were undertaking to prevent expiries or mitigate the effects of expiries. The most popular strategy (70/168, 41.7%) among both retailers and wholesalers was rational stocking, which involved stocking smaller amounts, stocking drugs with longer shelf lives, purchasing drugs that are popular with farmers and veterinarians, and buying drugs that are used to treat common diseases. Importantly, the strategies did not differ between retailers and wholesalers (*p* = 0.987). The details are shown in Table 6.

**Table 6:** Frequency of the strategies in place to prevent expiry of drugs in veterinary drug outlets in central Uganda

Strategy	Total	Retailer	Wholesaler
Discounts/lowering of prices	55 (32.7)	45 (81.8)	10 (18.2)
Rational stocking	70 (41.7)	57 (81.4)	13 (18.6)
Tracking stock	24 (14.3)	20 (83.3)	4 (16.7)
Promotional activities and increased marketing	19 (11.3)	15 (79.0)	4 (21.0)
Total	168 (100)	137 (81.5)	31 (18.5)

This table shows the strategies in place to prevent expiry of drugs among veterinary drug outlets studied. The most popular strategy (70/168, 41.7%) among both retailers and wholesalers was rational stocking which involved stocking smaller amounts, stocking drugs with longer shelf lives, purchasing drugs that are popular with farmers and veterinarians, buying drugs that are for treating common diseases

### Factors associated with expiry of veterinary medicines in supply outlets in central Uganda

A logistic regression analysis was conducted on all potential predictors of expiry of veterinary medicines. Bi-variable logistics regression analysis was used to obtain crude odds ratios (COR) and their respective 95% confidence intervals, while multi-variable logistics regression models were used to establish predictors of expiry of veterinary medicines. In the final analysis, the variables that remained statistically significant were sex, procurement policy, and practicing First-Expiry-First-Out.

Female in-charges were 2.9 times more likely to have an acceptable level of expiry of veterinary medicines, when compared to their male counterparts [adjusted OR = 2.9 (95% CI = 1.22–5.08, *p* = 0.017)]. Similarly, the odds of acceptable expiry levels of veterinary medicines were 3.9 times higher in outlets that possessed a procurement policy compared to the odds of those without a procurement policy [adjusted OR = 3.9 (95% CI = 1.29–4.81, *p* = 0.016)]. Still, the odds of acceptable expiry levels of veterinary medicines were six times higher in outlets that practiced First-Expiry-First-Out compared to the odds of those that did not practice this concept [adjusted OR = 6.07 (95% CI = 4.71–8.7, *p* = 0.002)]. The details are shown in Table 7.

**Table 7:** Logistic regression analysis of factors associated with the expiry of veterinary medicines in selected outlets in central Uganda

Variable	Categories	Level of expiry		Un adjusted model		Adjusted model	
		Acceptable *n*(%)	Unacceptable *n*(%)	COR (95% CI)	*p*-value	AOR (95% CI)	*p*-value
Sex	Male	44 (64.7)	24 (35.3)	Ref		Ref	
	Female	86 (84.0)	14 (14.0)	3.4 (1.58–4.11)	0.002	2.9 (1.22–5.08)	0.017*
Education level	Certificate	73 (74.5)	25 (25.5)	Ref			
	Diploma	37 (82.2)	8 (17.8)	1.6 (0.65–3.85)	0.311		
	Degree and above	20 (80.0)	5 (20.0)	1.37 (0.47–4.03)	0.568		
Supplier type	Retailer	4 (12.9)	27 (87.1)	Ref		Ref	
	Wholesaler/importer	34 (24.8)	103 (75.2)	2.23 (0.73–6.82)	0.161	2.74 (1.22–7.08)	0.106
Experience		Mean:5.2; median:4(SD4.2)		0.93(0.856–1.00)	0.060	0.94 (0.85–1.0)	0.197
Cost of stock	< 200 M	86 (75.4)	28 (24.6)	Ref			
	201 M–500 M	27 (84.4)	5 (15.6)	1.76 (0.62–5.00)	0.290		
	> 500 M	17 (77.3)	5 (22.7)	1.10 (0.37–3.27)	0.854		
Where the drugs are kept	Separate store	14 (73.7)	5 (26.3)	Ref			
	Both shelves and store	83 (77.6)	24 (22.4)	1.24 (0.40–3.78)	0.711		
	Outlet shelves	33 (78.6)	9 (21.4)	1.31 (0.37–4.61)	0.675		
Procurement policy	No	54 (62.8)	33 (37.2)	Ref		Ref	
	Yes	76 (92.7)	6 (7.3)	7.51 (2.93–9.20)	0.000	3.90 (1.29–4.81)	0.016*
First in first out	No	80 (87.9)	11 (12.1)	Ref		Ref	
	Yes	50 (64.9)	27 (35.1)	3.92 (1.79–8.61)	0.001	2.00 (0.73–5.53)	0.180
First expiry first out	No	123 (84.8)	22 (15.2)	Ref		Ref	
	Yes	7 (30.4)	16 (69.6)	12.8 (4.71–10.65)	0.000	6.07 (4.7–8.72)	0.002*
Electronic stock management tools	No	97 (76.4)	30 (23.6)	Ref			
	Yes	33 (80.5)	8 (19.5)	1.27 (0.53–3.06)	0.585		

This table shows the outputs of the logistic regression analysis for factors associated with the expiry of veterinary medicines in central Uganda. The variables sex; procurement policy; and practicing FEFO were statistically significant at 95% confidence interval

^*^Indicates that variables are statistically significant at a *p*-value ˂0.05

## Discussion

In this study, it was found that 88.1% of caretakers and/or owners of veterinary drug outlets had experienced expiries in their outlets in the past year. There were unacceptable levels of expiry in 22.6% of the outlets, and the majority of these were retail outlets. Additionally, most expiries occurred for drugs that had been supplied to the outlets on credit and those used to treat rare diseases. Irrational prescription; lack of accurate forecasting; overstocking of commodities; dry seasons; and stocking without considering stock-at-hand, were the major reasons that respondents cited as being responsible for the expiration. The main ways of disposal of expired drugs included throwing away at pits; burning at pits; returning them to suppliers; and incineration. The factors associated with acceptable levels of expiry included the caretaker being female, having a procurement policy, and practicing first expiry, first out.

In this study, expiries were fewer in outlets that were managed by those with higher education, i.e., wholesale outlets. In Uganda, the wholesale outlets are particularly pharmacies that are approved by the National Drug Authority (NDA). In the veterinary pharmacy approval guidelines, there is keen attention paid to a pharmacy having a supervising pharmacist, and these are degree holders [25]. It is a requirement in these guidelines that the supervising pharmacist be available full-time, unlike in drug shops, which also have restrictions on the types of veterinary drugs that they can stock. The stringent guidelines on pharmacies (who are usually wholesalers) are based on the fact that they are permitted to hold a wide range of classes of veterinary drugs that often necessitate strict professional handling. Because of this, the finding that most of the respondents held qualifications of a bachelor’s degree or above is in line with the existing NDA guidelines.

When it comes to the methods of inventory management in place, there are more retailers practicing both First-In-First-Out (FIFO) and First-Expired-First-Out (FEFO) than wholesalers, who are doing more of the latter than the former. This is used in pharmacy, specifically in the area of inventory. In FIFO, first in medication and supplies are taken out first and many times, stock must be rotated so that the first items in must be dispensed first. Some authors have noted that this can be difficult, especially for those that stock large amounts of goods [27]. The difficulty comes because, in most cases, the new stock goes behind current stock on the shelves. This may call for moving and rearranging the current inventory in order to place the new, ordered product in the back. This may explain why the wholesalers rely on the FEFO method because of the larger capacities that they hold. Besides, as can be noted from the findings, wholesalers are more inclined to use electronic inventory management systems than retailers. Therefore, they can use FEFO with some degree of ease.

In this study, 22.6% of the drug outlets had experienced unacceptable levels of drug expiry and approximately 90% of these were retailers. The finding that more expiries are at the retail level is in line with the other findings of the study where the respondents cited reasons for expiry being drugs with shorter shelf lives. Earlier studies conducted in Uganda and Ethiopia also had similar findings, where short shelf life, inaccurate forecasting of needs, bad storage practices, and inadequate inventory controls were identified as major factors for high medicine wastage, albeit on the human side [17, 28]. However, it should be noted that the earlier studies were located in similar settings which may explain the similarity of findings. This means that both retailers and wholesalers need to check the shelf lives of veterinary drugs, especially those that have been identified as having high wastage rates attributed to expiration. In this study, these have been identified as powders (for mainly treating poultry diseases) and antibiotic injectables. These should be closely monitored or even shifted to the front of the shelf or reallocated to business branches that have higher consumption rates for those medicines.

The respondents identified the drugs that expire most as those that are usually supplied on credit from the wholesalers. Whereas little literature exists on the association between credit supplies and goods expiry, in the local context of Uganda, suppliers tend to push nearly expiring drugs out to the retailers in the hope for faster sales. In the process, the retailers also stock such goods in larger amounts than they need to, resulting in overstocking. Further, the finding that drugs that expire are those that are used to treat rare diseases or conditions is in agreement with a previous study on human medicines in Uganda [17]. Such drugs tend to be expensive, thus the slow turnover. This is aggravated by the conditions they are used for being rare and therefore less prescribed.

That inaccurate forecasts were a key reason for expiries in this study is not surprising. This is in agreement with a study in Ethiopia, which identified weak monitoring systems along supply chains as a key cause of inaccurate forecasts [28]. In line with this, previous researchers have emphasized that one of the most important keys to succeeding in supply chain management is demand forecasting [29]. If the demand forecasting is incorrect, it triggers a multitude of problems across the entire supply chain. To make it worse, various authors have pointed out that existing bottlenecks in the supply chain systems for medicines obstruct the accurate forecasting of demand, and with the inability to forecast demand with certainty, the stakeholders cannot plan and make commitments for the future [30]. Therefore, the actors in the supply chain of veterinary medicines in Uganda need to devise ways of improving the forecasting actions, both in frequency and quality.

Dry seasons were also cited as a major contributor to drug expiries. This may be explained by the works of past authors who have decried the effects of dry seasons on the livestock sub-sector. During the dry seasons, there are challenges on livestock production such as reduction in general performance of animals, increased susceptibility to diseases, reduction in palatability and acceptability of available forage, reduction in digestibility of forage consumed, migration of flock and herd’s men, overcrowding of available graze land, sales of animals at loss and increased cost of production [31]. Furthermore, a sizable number of Uganda’s livestock owners are pastoralists. These have been cited to suffer losses in production that are manifested in reduced monetary value of animals and deaths during severe droughts [32]. All these factors will impede sales of veterinary products, which results in economic losses for suppliers.

The respondents in this study also decried the irrational use of some veterinary medicines as one of the causes of expiries. Rational use of medicines is when patients (in this case, animals) obtain medications suitable for their clinical needs, in doses that are in line with their specific individual requirements, for a satisfactory period of time, and at the lowest cost to them and the broader community [33]. Indeed, the World Bank divided rational use of drugs into two aspects: the use of drugs according to scientific data on efficacy, safety, and compliance; and the cost-effective use of drugs within the constraints of a given health system [34, 35]. In other settings, factors related to irrational use of drugs have been described in various studies, including erroneous diagnosis, insufficient awareness and information, low experience, asymmetry of information, poor medical education, ineffective monitoring, inadequate regulation of prescriptions, and use of medicines that are either nearing expiry dates or are already expired [36]. Inappropriate and irrational use of veterinary drugs has been widely reported in Uganda [37–39] as well as Ethiopia [40], settings that are similar to our study area. Therefore, it is not surprising that a significant number of respondents mentioned it as one of the key reasons for the expiries in their outlets.

Poor stock monitoring and lack of data on stock were cited as some of the reasons for the wastage through expiries. This is in consonance with previous studies that have cited the same in Uganda and South Africa [17, 41]. The similarities may be explained by the fact that automated logistics management information systems were not widely used by the respondents in the two comparative studies, even when they were available in South Africa. The wholesalers and retailers must therefore keep up-to-date inventory records. Proficient management of the drug outlet and good control of stock habitually guarantee that the correct medicine is available at the right place and time while not typing up capital unnecessarily, resulting into expiries. It also protects against many other glitches arising in the supply chain [42].

In this study, 54% of respondents said they throw expired medicines in pits or burn them. There fact that such environmentally non-friendly practices by businesses are taking place is worrying. Veterinary professionals have indicated that medicinal waste disposal is a significant part of healthcare waste management within a veterinary practice. This is why it must be incorporated into the scheme of work for veterinary medical waste management; and the rules and requirements across the waste management requirements within the practice [43]. When veterinary businesses deviate from this in Uganda, there are several risks. For example, the Toxic Substances Hydrology Program of the United States Geological Survey reports that some water samples analyzed in the US had a broad range of chemicals from human and veterinary drugs, among other household, industrial, and agricultural chemicals [44].

With regard to the environment, corporate social responsibility (CSR) and sustainability management have received increasing attention from researchers and practitioners over the past decades [45]. This calls for a balanced approach to their environmental, social, and economic performance, although recent developments in business focus more on environmental responsibility [46]. By doing so, businesses have demonstrated that they improve their financial performance. Besides, studies identified building green image as a tool to help firms enhance their green competitive capacity and therefore competitive advantage [47]. Therefore, by continuing with environmentally unfriendly practices, the veterinary drug outlets in Uganda not only endanger public health but also miss out on global business trends and hence profitability. However, at 30%, the return-to-supplier practice is at a higher and better rate than in the USA, where take-back programs were the most common disposal method at 22% [48]. The differences in the proportions for the two studies may be explained by the abundance of other means of disposal in the USA. Besides, in the United States, few take-back programs have been reported as being successful [48].

In order to prevent expiries, the respondents in this study cited using different strategies, including offering discounts or lowering prices, rational stocking, tracking stock, promotional activities, and increased marketing. The finding of using discounts in pharmaceutical businesses to reduce the expiration of inventory has been reported in other studies [49]. This is always done in the hope that lower prices will drive demand for such products. Coupled with promotional activities for such products to create awareness and increase demand, the respondents found demand-driven stocking an effective strategy in prevention of expiration of veterinary drugs.

The veterinary drug outlets run by females were 2.9 times more likely to have an acceptable level of expiry of veterinary medicines when compared to the male practitioners. Much as this is a good finding, the authors could not establish the reasons behind it, and no study was available to attest to it. However, some authors have found females to be more meticulous and better at handling different work tasks at the same time than males [50]. Nevertheless, the odds of acceptable expiry levels of veterinary medicines were 3.9 times higher in outlets that possessed a procurement policy compared to the odds of those without a procurement policy. A procurement policy is a tool that guides the stocking of a veterinary pharmacy, which is why its presence should not be discounted.

The procurement policy establishes procedures for the business for the procurement of all goods and services and ensures that all goods and services procured are obtained at cost-effective prices, meet the required specifications and quality, and are delivered on time [51]. It such a policy that ensures procuring quality goods and services from reliable and well-established suppliers; effective negotiations with the suppliers to obtain quantity discounts; continuous investigations on new suppliers and market prices trend goods and services the business continuously requires; procuring prudently by placing emphasis on competition and selection of vendors whose offers confirms to the terms and conditions as well as the technical requirements and specifications stated on purchase requisitions; and management of stock inventory to provide best service to users [52]. Given such benefits, it is not surprising that veterinary drug outlets that had them were performing well when it came to expiries.

In this study, the odds of acceptable expiry levels of veterinary medicines were six times higher in outlets that practiced First-Expiry-First-Out compared to the odds among those that did not practice this concept. The findings of this study are in consonance with those of earlier authors who recommended that, for developing countries, pharmaceutical businesses must ensure a system of stock usage based on expiration dates to ensure appropriate stock rotation [26]. In the human pharmaceutical industry, this strategy is commonly used in Uganda to prevent stock expirations [17].

The findings of this study have implications at both the business and policy levels. Rampant expiration of veterinary drugs translates into loss of income for businesses. This means that business practices and human resources should be reoriented to incorporate effective measures of expiry mitigation, such as supply chain monitoring. This will help them implement demand-driven stocking based on more efficient forecasting. At the policy level, there is a need to protect animals, animal product consumers, and the environment from the effects of poor disposal of expired veterinary drugs. This can only be done if expired veterinary drugs and their effects are incorporated as an important component of surveillance systems for animal, human, and environmental health in Uganda.

The limitation to this study was that some of the variables, e.g., what the respondents thought were causes of expiries from their perspective, were subjective measures, and respondents may not have been uniform because of individual variations. In addition, for some variables, the responses were dependent on the respondent with no means of verification. The data collectors were encouraged to stick to the questionnaire as well as ask triangulation questions where possible to verify the responses.

## Conclusions

The expiry of veterinary drugs is still high, especially in retail outlets, and this is attributed to irrational prescription, lack of accurate forecasting, overstocking of commodities, dry seasons, lack of adequate data on stock, and the procurement of commodities irrespective of the available stock. Additionally, the disposal of expired drugs does not often follow environmentally friendly approaches. Further, having a written procurement policy and practicing the concept of FEFO is a good way of preventing the expiry of veterinary drugs. We recommend that supply chain actors embrace a multi-disciplinary approach in the prevention of drug expiries, for example, by making data-driven decision of stocking in a wider perspective from demand to weather forecasts to accommodate the dry seasons. In addition, environmental preservation agencies like the National Environmental Management Authority (NEMA) should empower veterinary drug outlets to follow appropriate methods of disposal of expired drugs. Dealers should also be empowered with skills and technologies that deter expiries, and practitioners should adopt rational methods of prescription in order to prevent drug misuse and the expiry of otherwise efficacious veterinary drugs.

## Data Availability

Available on request.

## Abbreviations

FEFO: First-expire-first-out
FIFO: First-in-first-out
GDP: Gross domestic product
IQR: Interquartile range
NDA: National Drug Authority
NEMA: National Environmental Management Authority
SDG: Sustainable Development Goal

## References

[1] Bar-On YM, Phillips R, Milo R (2018). The biomass distribution on Earth. Proc Natl Acad Sci.

[2] Herrero M (2012). The roles of livestock in developing countries. Animal Int J Anim Biosci.

[3] Salmon GR (2020). Exploring the landscape of livestock ‘Facts’. Glob Food Sec.

[4] Thornton P, Herrero M. Climate change, vulnerability and livestock keepers: challenges for poverty alleviation. In: Rowlinson P, Steele M, Nefaoui A, editors. Livest and Glob Clim Change: Proceedings of the Livestock and Global Climate Change Conference, Hammamet, 17-20 May 2008, Cambridge, UK: CUP. 2008; p. 21–24.

[5] Upton M. The Role of Livestock in Economic Development and Poverty Reduction. PPLPI Working Papers 23783, Food and Agriculture Organization of the United Nations, Pro-Poor Livestock Policy Initiative. 2004. 10.22004/ag.econ.23783.

[6] Vudriko P, Kikomeko R, Majalija S. Evaluating pricing and affordability of veterinary drugs among livestock farmers: A case study of Mityana district in central Uganda. Livest Res Rural Dev. 2011;23(7):Article #162. Retrieved April 4, 2021, from http://www.lrrd.org/lrrd23/7/vudr23162.htm.

[7] Grace D, Songe M, Knight-Jones T. Impact of neglected diseases on animal productivity and public health in Africa. Presentation at the 21st conference of the World Organisation for Animal Health (OIE) regional commission for Africa, Rabat, Morocco, 16-20 February 2015. Nairobi: ILRI. 2015.

[8] Tongue LK, Ngapagna AN. Emerging vector-borne diseases in Central Africa: a threat to animal production and human health. In: Claborn D, Bhattacharya S, Roy S, editors. Current topics in the epidemiology of vector-borne diseases. London: Intech Open; 2019. p. 1–14.

[9] Grasswitz T, et al. The veterinary pharmaceutical industry in Africa: a study of Kenya, Uganda and South Africa. African Union/Interafrican Bureau for Animal Resources (AU/IBAR), Nairobi, Kenya, 2004.

[10] Taremwa F. Assessment of knowledge, attitude and disposal practices of unused and expired veterinary pharmaceuticals among farmers, veterinary practitioners and drug shop operators in Ibanda District. 2022, Makerere University.

[11] Dione MM (2021). Supply chain and delivery of antimicrobial drugs in smallholder livestock production systems in Uganda. Front Vet Sci.

[12] Freitas LAA, Radis-Baptista G (2021). Pharmaceutical pollution and disposal of expired, unused, and unwanted medicines in the Brazilian. Context.

[13] Alaska Department of Environmental Conservation. Prescription and veterinary medicine disposal. Division of Environmental Health 2022. https://dec.alaska.gov/eh/solid-waste/how-do-i-dispose-of/prescription-and-vet-medicine/. Accessed 18 Feb 2023.

[14] National Drug Authority, A guide to disposal of pharmaceutical waste. 2022, National Drug Authority of Uganda.

[15] National Drug Authority. Drug shops licensed. 2020. https://www.nda.or.ug/drug-shops-licensed-in-2020/. Accessed 18 Feb 2023.

[16] Kamba PF (2017). Threats posed by stockpiles of expired pharmaceuticals in low-and middle-income countries: a Ugandan perspective. Bull World Health Organ.

[17] Nakyanzi JK (2010). Expiry of medicines in supply outlets in Uganda. Bull World Health Organ.

[18] Wangmo K (2021). Knowledge, attitude, and practice on antibiotic use and antibiotic resistance among the veterinarians and para-veterinarians in Bhutan. PLoS ONE.

[19] Kainga H (2023). Determinants of knowledge, attitude, and practices of veterinary drug dispensers toward antimicrobial use and resistance in main cities of Malawi: a concern on antibiotic stewardship. Antibiotics.

[20] Desta AH (2015). Veterinary drugs handling, management and supply chain assessment in Afar pastoral region of North East Ethiopia. Am J Biosci Bioeng.

[21] Saman A (2023). Assessment of knowledge, perception, practices and drivers of antimicrobial resistance and antimicrobial usage among veterinarians in Pakistan. Prev Vet Med.

[22] Lam J (2018). Environmental stewardship practices of veterinary professionals and educators related to use and disposal of pharmaceuticals and personal care products. J Am Vet Med Assoc.

[23] Oliveira KSd (2020). Disposal of animal healthcare services waste in southern Brazil: One Health at risk. Saúde em Debate..

[24] The World Bank Group (2017). The role of city governments in economic development of greater Kampala.

[25] National Drug Authority (2018). Licensing Guidelines 2019.

[26] Tull K. Drug expiry standards in developing countries. K4D Brighton: Institute of Development Studies. 2018. p. 1–18.

[27] Asim M. What is FIFO as it relates to the Retail Pharmacy Industry? 2020. https://www.pharmacy-tech-resources.com/what-is-fifo-as-it-relates-to-the-retail-pharmacy-industry.html. Accessed 22 Sept 2021.

[28] Gebremariam ET, Gebregeorgise DT, Fenta TG (2019). Factors contributing to medicines wastage in public health facilities of South West Shoa Zone, Oromia Regional State, Ethiopia: a qualitative study. J Pharm Policy Pract.

[29] Al-Zaidi WA, Al-Karawi AJ, Al-Zuhairi AK. Role of demand forecasting and lead time on waste in supply chain: a case study in Diyala Health Sector-Iraq. J Adv Manage Sci. 2018;6(3):155–160. 10.18178/JOAMS.6.3.155-160.

[30] Subramanian L (2021). Effective demand forecasting in health supply chains: emerging trend, enablers, and blockers. Logistics.

[31] Lamidi A, Ologbose F (2014). Dry season feeds and feeding: a threat to sustainable ruminant animal production in Nigeria. J Agric Soc Res (JASR).

[32] Ndathi AJ, Nyangito MN, Musimba NK, Mitaru BN. Climate variability and dry season ruminant livestock feeding strategies in Southeastern Kenya. Livest Res Rural Dev. 2011;23(9). Retrieved May 10, 2021, from http://www.lrrd.org/lrrd23/9/ndat23199.htm.

[33] World Health Organization. Conference of experts on the rational use of drugs. World Health Organization, Nairobi, Kenya, WHO/CONRAD/WP/RI,(25-29.12. 1985). 1985. Retrieved May 10, 2021, from https://apps.who.int/iris/handle/10665/62311.

[34] Almarsdóttir AB, Traulsen JM (2005). Rational use of medicines–an important issue in pharmaceutical policy. Pharm World Sci.

[35] Ofori-Asenso R, Agyeman AA (2016). Irrational use of medicines—a summary of key concepts. Pharmacy (Basel, Switzerland).

[36] Mohamadloo A, Ramezankhani A, Zarein-Dolab S, Salamzadeh J, Mohamadloo FA. Systematic review of main factors leading to irrational prescription of medicine. Iran J Psychiatry Behav Sci. 2017;11(2):e10242. 10.5812/ijpbs.10242.

[37] Basulira Y, Olet SA, Alele PE (2019). Inappropriate usage of selected antimicrobials: comparative residue proportions in rural and urban beef in Uganda. PLoS ONE.

[38] Nayiga S (2020). Use of antibiotics to treat humans and animals in Uganda: a cross-sectional survey of households and farmers in rural, urban and peri-urban settings. JAC-Antimicrob Resist..

[39] McCubbin KD (2021). Unsafe “crossover-use” of chloramphenicol in Uganda: importance of a One Health approach in antimicrobial resistance policy and regulatory action. J Antibiot.

[40] Beyene T, Tesega B (2014). Rational veterinary drug use: its significance in public health. J Vet Med Anim Health.

[41] Mahoro A. Examining the inventory management of antiretroviral drugs at community health centres in the Cape Metropole, Western Cape. Thesis, University of the Western Cape. 2013. p. 1–96.

[42] Rock A, Ackerman N (2010). Practical notes on pharmacy management: part 1. Vet Nurs J.

[43] BSAVA. BSAVA guide to the use of veterinary medicines. British Small Animal Veterinary Association; 2020.

[44] Kolpin DW (2002). Pharmaceuticals, hormones, and other organic wastewater contaminants in US streams, 1999–2000: a national reconnaissance. Environ Sci Technol.

[45] Galbreath J (2010). Drivers of corporate social responsibility: the role of formal strategic planning and firm culture. Br J Manag.

[46] Lee K-H, Cin BC, Lee EY (2016). Environmental responsibility and firm performance: the application of an environmental, social and governance model. Bus Strateg Environ.

[47] Alam SMS, Islam KMZ (2021). Examining the role of environmental corporate social responsibility in building green corporate image and green competitive advantage. Int J Corp Soc Responsib.

[48] Vatovec C (2021). Pharmaceutical pollution sources and solutions: survey of human and veterinary medication purchasing, use, and disposal. J Environ Manage.

[49] Bardeji SF (2020). Perishable inventory management using GA-ANN and ICA-ANN. Int J Procure Manage.

[50] Szameitat AJ (2015). "Women are better than men"—public beliefs on gender differences and other aspects in multitasking. PLoS ONE.

[51] Sanlam. Procurement policy and procedures 2018, Sanlam: Nairobi, Kenya.

[52] Bower AG. Procurement policy and contracting efficiency. Int Econ Rev. 1993;34(4):873–901.

